# Hemosiderin induced acute kidney injury requiring hemodialysis in patients with paroxysmal nocturnal hemoglobinuria: A case report

**DOI:** 10.1097/MD.0000000000035412

**Published:** 2023-10-06

**Authors:** Heejung Choi, Hyunee Yim, Min-Jeong Lee

**Affiliations:** a Department of Nephrology, Ajou University School of Medicine, Suwon, Korea; b Department of Pathology, Ajou University School of Medicine, Suwon, Korea.

**Keywords:** acute kidney injury, acute tubular necrosis, case report, hemolysis, paroxysmal nocturnal hemoglobinuria

## Abstract

**Rationale:**

Paroxysmal nocturnal hemoglobinuria (PNH) is a rare hematopoietic stem cell disease with features of hemolytic anemia, thrombosis, and bone marrow failure. Due to intravascular hemolysis and hemoglobinuria, renal dysfunction is often accompanied in PNH patients.

**Patient concerns:**

A 25-year old woman presenting gross hematuria after coronavirus disease 2019 infection was admitted to our medical center. She had mild nausea and headache. She was diagnosed with iron deficiency anemia few years ago and had no other underlying disease. Her laboratory findings showed acute kidney injury (AKI) and severe anemia, with evidences of hemolysis.

**Diagnosis:**

Renal biopsy was done to determine the cause of renal failure and the result was acute tubular necrosis with deposition of golden pigments, hemosiderin. With pathologic result and laboratory finding of hemolysis, we did flow cytometry for PNH, and the patient was finally diagnosed with PNH.

**Interventions:**

With AKI and oliguria, the patient started to take hemodialysis.

**Outcomes:**

After taking 5 sessions of hemodialysis, the patient’s renal function was recovered from AKI. With diagnosis of PNH, the patient is now being treated with complement C5 inhibitor.

**Lessons:**

This challenging case tells us that we should consider the first manifestation of PNH as a cause of severe AKI requiring hemodialysis in a patient with anemia and evidence of hemolysis.

## 1. Introduction

Paroxysmal nocturnal hemoglobinuria (PNH) is a rare hematopoietic stem cell disease with features of hemolytic anemia, thrombosis, and bone marrow failure.^[1]^ Renal dysfunction is also a known clinical characteristic of PNH, which can be manifested as acute kidney injury (AKI) or chronic kidney disease as a result of hemolysis.^[2]^ We report a patient who occurred acute tubular necrosis (ATN) with coronavirus disease 2019 (COVID-19) infection, and was finally diagnosed with PNH. The patient had provided informed consent for publication of the case.

## 2. Case presentation

A 25-year old woman was admitted to nephrology department due to AKI. Four days ago, she was confirmed with COVID-19 infection. From the next day, gross hematuria was started, so she was hospitalized in Suwon Hospital on the third day of COVID-19 infection. Her laboratory tests at the time of admission to Suwon Hospital showed white blood cells (WBC) 3.57*10^3^/μL, red blood cells (RBC) 2.18*106/μL, hemoglobin 5.5 g/dL, platelet 101*10^3^/μL, prothrombin time 11.6 seconds, activated partial thromboplastin time 24.5 seconds, blood urea nitrogen (BUN) 79.4 mg/dL, creatinine 8.31 mg/dL, estimated glomerular filtration rate (calculated by modification of diet in renal disease equation) 5.87 mL/min/1.7 m^2^, lactate dehydrogenase (LDH) 6260 U/L, total bilirubin 2.6 mg/dL, aspartate transaminase 250 U/L, alanine transaminase 41 U/L. Urine analysis showed dark-amber color, with specific gravity 1.012, pH 6.5, leukocyte esterase 2+, nitrite 2+, protein 1+, blood 2+. Urine microscopy showed RBC 101 to 200/high-power field (HPF) and WBC > 200/HPF. Although she was managed with transfusion of packed RBC, hydration, and diuretics, her urine output was not recovered and serum creatinine continued to increase. On the next day, she was transferred to tertiary hospital considering start of renal replacement therapy and renal biopsy.

She had alert mental status with acute ill looking appearance when she arrived in our hospital. At the time of admission, blood pressure was 128/84 mmHg, pulse rate was 72 beats per minute, and body temperature was 36.9 °C. She had mild nausea and headache. On physical examination, her lungs, heart, abdomen and nervous system were normal. She said that she was diagnosed with iron deficiency anemia in another hospital several years ago, and she remembered that her hemoglobin level was about 7.0 mg/dL at that time. She had no other underlying disease except anemia, and she had not been exposed to any nephrotoxic agent recently. Her laboratory tests showed WBC 2.8*10^3^/μL, RBC 2.56*10^6^/μL, hemoglobin 7.0 g/dL, platelet 94*10^3^/μL, mean corpuscular volume 85.4 fL, mean corpuscular hemoglobin 27.4 pg, mean corpuscular hemoglobin concentration 32.1 g/dL, and reticulocyte count 1.84%. Peripheral blood smear showed normocytic hypochromic anemia with anisocytosis, poikilocytosis, and schistocytes. Anemia study showed serum iron 33 ug/dL, total iron binding capacity 275 ug/dL, ferritin 576 ug/L, and haptoglobin < 10 mg/dL. Coagulation tests showed prothrombin time 13.0 seconds and activated partial thromboplastin time 38.0 seconds. Serum biochemistry showed blood urea nitrogen 87.6 mg/dL, creatinine 9.70 mg/dL, estimated glomerular filtration rate (modification of diet in renal disease equation) 4.91 mL/min/1.7 m^2^, LDH 2146 U/L, total bilirubin 1.4 mg/dL, direct bilirubin 0.8 mg/dL, aspartate transaminase 141 U/L, alanine transaminase 41 U/L, and C-Reactive protein 5.88 mg/dL. Urinalysis showed amber color, specific gravity 1.009, pH 6.5, WBC ester 3+, nitrite -, protein 2+, blood 4+, glucose −, ketone −, bilirubin −, and urobilinogen <1.0. Urine microscopy showed many RBCs/HPF and many WBCs/HPF. Serum antinuclear antibodies, anti-double stranded DNA antibodies, anti-neutrophil cytoplasmic antibodies, anti-glomerular basement membrane antibodies, and rheumatoid factor were all negative. With signs of hemolysis like elevated LDH, decreased haptoglobin, and schistocytes, we performed tests about hemolytic anemia. Direct and indirect antiglobulin test were negative. A disintegrin and metalloproteinase with a thrombospondin type 1 motif, member 13 activity was 44.4%, and E.coli O157:H7 was negative. CH50 was 47.8 U/mL, within normal range. Ultrasound image showed normal echogenicity of both kidneys with length of 12.6 cm of right kidney and 13.2 cm of left kidney. There was no evidence of hydronephrosis.

**Figure 1.**
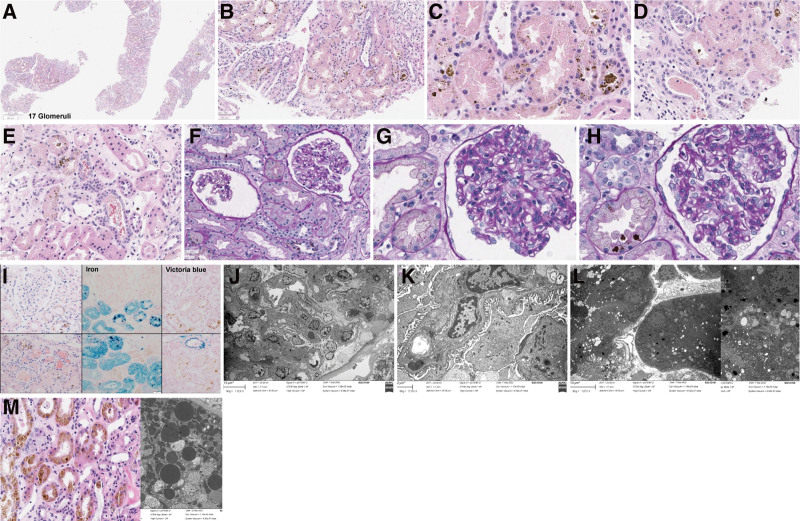
Renal biopsy.

During the first 3 hospital days, her urine output was 880 mL, 400 mL, and 600 mL respectively. Her serum creatinine increased to 13.97 mg/dL. Finally, she started hemodialysis on hospital day 4, after her isolation period of COVID-19 was over. On hospital day 5, we performed renal biopsy to identify the cause of AKI (Fig. 1). On light microscopy, all glomeruli looked normal in size and cellularity, and no necrotic lesion or thrombosis was present in the glomeruli. Glomerular capillary walls were thin and even, and there was no segmental sclerosis. Tubules occasionally showed eosinophilic necrotic debris. Tubular epithelial cells frequently revealed golden brown, refractile pigments, which were positive for iron stain. Blood vessels were unremarkable. On electron microscopy, ultrastructural features of a glomeruli were unremarkable. Final report of renal biopsy was ATN with a comment that deposition of golden pigments, hemosiderin can be observed in intravascular hemolysis such as hemolytic anemia or mechanical heart valves.

Before renal biopsy result was reported, we considered the possibility of thrombotic microangiopathy like atypical hemolytic uremic syndrome with evidence of hemolysis, thrombocytopenia, and elevated creatinine. However, with final report of ATN on renal biopsy, we considered possibility of PNH. We performed flow cytometry for PNH. Finally, the patient was diagnosed with PNH.

During hemodialysis session, the patient complained of nausea and headache, but it improved over time. After 5 times of hemodialysis, the patient’s urine output started to increase and serum creatinine started to decrease. As her renal function recovered over time, the patient felt that her general condition improved. She stopped to take hemodialysis and discharged on hospital day 13.

**Figure 2.**
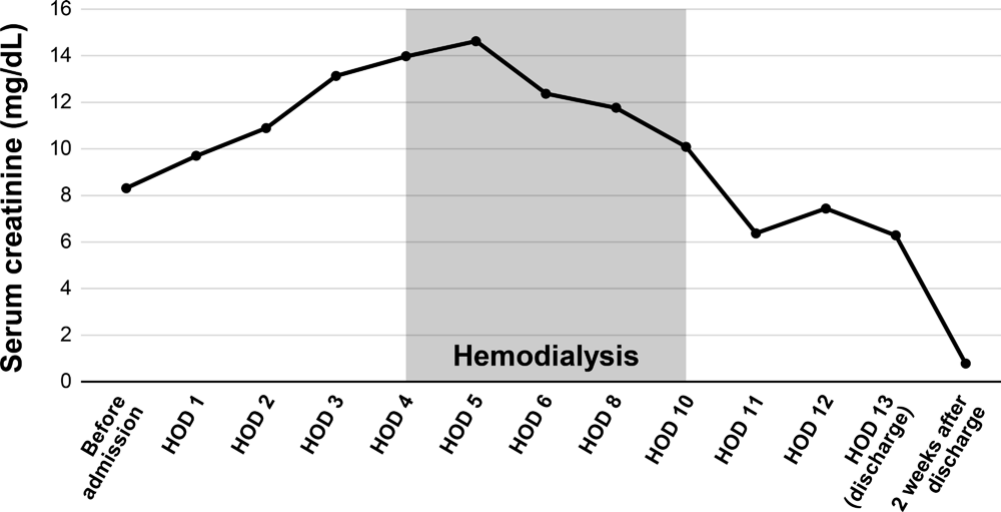
Changes of clinical parameters of serum creatinine.

**Figure 3.**
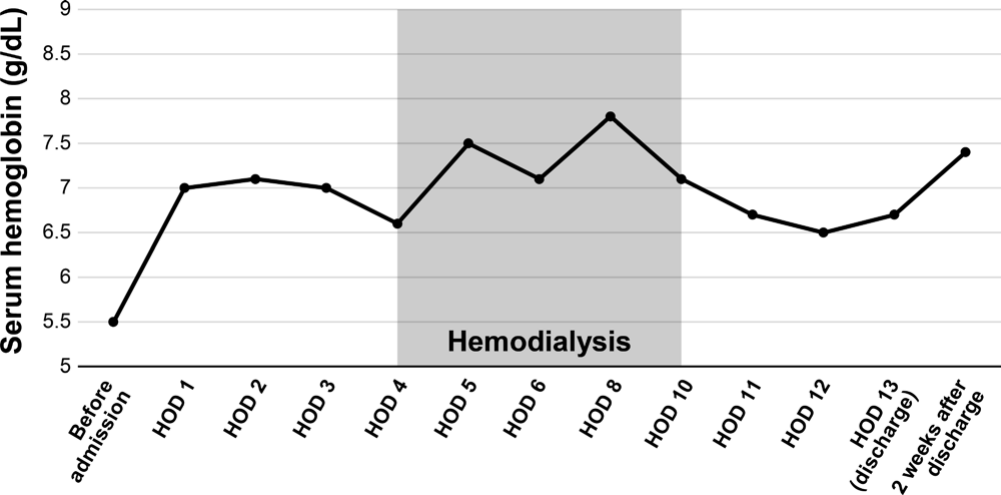
Changes of clinical parameters of serum hemoglobin.

**Figure 4.**
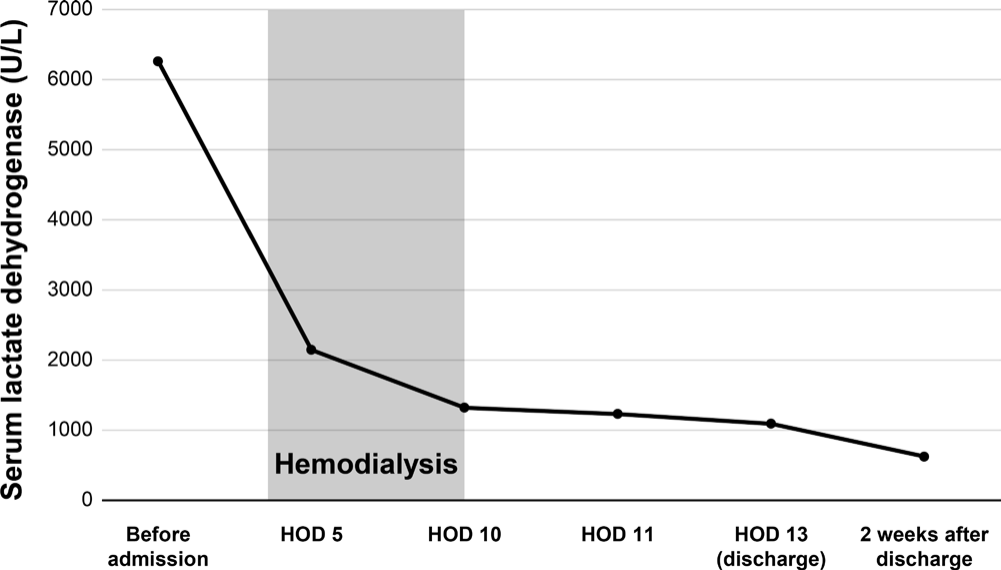
Changes of clinical parameters of serum lactate dehydrogenase.

She visited outpatient clinic after 2 weeks and her serum creatinine was 0.78 mg/dL, fully recovered from ATN (Figs. 2–4). After the diagnosis of PNH, she is now receiving ravulizumab, a complement C5 inhibitor.

## 3. Discussion

PNH is a rare clonal hematopoietic stem cell disease that acquires a mutation in a gene called phosphatidylinositol glycan anchor biosynthesis, class A. This results in a reduction or absence of glycosylphosphatidylinositol anchor proteins. CD55 and CD59 are glycosylphosphatidylinositol-anchored proteins and lack of these proteins in PNH leads to complement mediated intravascular hemolysis.^[3]^ Although hemolysis, pancytopenia, and thrombosis are well known triad of clinical features of PNH, renal dysfunction is also a frequent clinical characteristic observed in PNH, and it can be acute or chronic.^[2,4]^ There has been several studies that reported AKI as the first clinical sign in PNH.^[5–7]^ Also, 1 study reported that up to 65% of PNH patients present chronic kidney disease (stages 1–5), and 21% of PNH patients have chronic kidney disease stages 3 to 5.

Chronic renal failure can be a result of chronic hemolysis due to a continuous state of complement activation in PNH. Repeated exposure of hemoglobin to renal epithelium can lead to intrarenal deposition of hemosiderin, and biopsy findings have shown hemosiderin deposition in proximal tubules.^[2,7]^

Likewise, acute massive hemolysis can cause AKI in PNH with various mechanisms. Hemoglobin has direct toxicity to renal tubules, and this toxicity is proven to be dose-dependent, which means AKI is likely to occur when hemolysis is sudden and massive.^[7]^ Also, hemolysis induce heme pigment casts or uric acid crystals, and they obstruct renal tubules.^[8]^ In addition, large amount of free hemoglobin produced by hemolysis binds to nitric oxide (NO) and serves as a potent scavenger, inducing intrarenal vasoconstriction.^[3]^ With these mechanisms, acute massive hemolysis result in AKI. Acute episodes of hemolysis can take place in situations like infection, transfusion, surgery, strenuous activity, and excessive alcohol intake in PNH.^[3,7]^

Another mechanism associated with renal impairment is hypercoagulable tendency in PNH. Thrombosis is a common complication of PNH, occurring in 40% of PNH patients.^[1]^ Gross renal vessel thrombosis can occur, but renal ischemia can result from recurrent microvascular thrombosis.^[1]^ This microvascular thrombosis lead to ischemic necrosis of renal cortex.

In our case, a young woman, who was diagnosed with anemia several years ago, developed AKI with COVID-19 infection, and finally diagnosed with PNH. It seems like chronic hemolysis due to PNH was already going on years ago when her anemia was firstly recognized, and COVID-19 infection triggered acute hemolytic episode. Since infection itself is a common cause of AKI and the patient told us that she was previously diagnosed with iron deficiency anemia, it was not easy to think of PNH as cause of AKI. However, her hemoglobin level was too low and did not respond to transfusion or erythropoietin. Also, there were evidences of hemolysis, like increased LDH, low haptoglobin, and schistocytes. Therefore, we could consider the possibility of PNH in this patient. We should always think of PNH as a possible cause of AKI in patients with unexplained anemia.

## Author contributions

**Investigation:** Heejung Choi, Min-Jeong Lee.

**Project administration:** Min-Jeong Lee.

**Resources:** Hyunee Yim.

**Supervision:** Min-Jeong Lee.

**Visualization:** Heejung Choi.

**Writing – original draft:** Heejung Choi.

**Writing – review & editing:** Hyunee Yim, Min-Jeong Lee.

## References

[1] Devalet B Mullier F Chatelain B. Pathophysiology, diagnosis, and treatment of paroxysmal nocturnal hemoglobinuria: a review. Eur J Haematol. 2015;95:190–8. 25753400 10.1111/ejh.12543

[2] Kokoris SI Gavriilaki E Miari A. Renal involvement in paroxysmal nocturnal hemoglobinuria: an update on clinical features, pathophysiology and treatment. Hematology. 2018;23:558–66. 29486674 10.1080/10245332.2018.1444563

[3] Pu JJ Brodsky RA. Paroxysmal nocturnal hemoglobinuria from bench to bedside. Clin Transl Sci. 2011;4:219–24. 21707954 10.1111/j.1752-8062.2011.00262.x PMC3128433

[4] Hillmen P Elebute M Kelly R. Long-term effect of the complement inhibitor eculizumab on kidney function in patients with paroxysmal nocturnal hemoglobinuria. Am J Hematol. 2010;85:553–9. 20658586 10.1002/ajh.21757

[5] Ballarín J Arce Y Torra Balcells R. Acute renal failure associated to paroxysmal nocturnal haemoglobinuria leads to intratubular haemosiderin accumulation and CD163 expression. Nephrol Dial Transplant. 2011;26:3408–11. 21771756 10.1093/ndt/gfr391

[6] ChenSCHungCCHsuCP. Recurrent acute renal failure in a patient with aplastic anemia-paroxysmal nocturnal hemoglobinuria syndrome: a case report. Kaohsiung J Med Sci. 2007;23:579–83.18055307

[7] Chow KM Lai FM Wang AY. Reversible renal failure in paroxysmal nocturnal hemoglobinuria. Am J Kidney Dis. 2001;37:E17. 11157403 10.1053/ajkd.2001.21361

[8] Wijewickrama ES Gooneratne L De Silva C. Acute tubular necrosis in a patient with paroxysmal nocturnal hemoglobinuria. Saudi J Kidney Dis Transpl. 2013;24:105–8. 23354203 10.4103/1319-2442.106302

